# Comparing Two Early Child Development Assessment Tools in Rural Limpopo, South Africa

**DOI:** 10.1186/s12887-020-02101-0

**Published:** 2020-05-07

**Authors:** Gwyneth Milbrath, Claire Constance, Audrey Ogendi, James Plews-Ogan

**Affiliations:** 1 University of Illinois Chicago College of Nursing, 845 S Damen Ave MC802, Chicago, IL 60612 USA; 2 Fors Marsh Group, Arlington, USA; 3 Michigan State University, Lansing, USA; 4 University of Virginia Children’s Hospital, Charlottesville, USA

**Keywords:** early child development, developmental assessment, South Africa, rural health, public health nursing

## Abstract

**Background:**

Providing increased cognitive stimulation or learning opportunities to young children significantly increases cognitive and social-emotional competence later in life. This study aims to determine the acceptability of a pediatric assessment tool to track early child development (ECD) in a rural health district in Limpopo, South Africa.

**Methods:**

A total of 11 primary health nurses from the region in two focus groups were selected to learn and compare two ECD assessment tools: the Cognitive Adaptive Test/Clinical Linguistic and Auditory Milestone Scale (CAT/CLAMS) and Ages and Stages Questionnaire (ASQ). Data were analyzed using versus coding to compare between the two focus groups and between ASQ and CAT/CLAMS.

**Results:**

The major categories that emerged from the discussion were current practice, usability, resource management, cultural adaptation, patient and parent factors, and new knowledge.

**Conclusions:**

This study illustrates the challenges related to adapting and implementing ECD assessment in an environment where ECD is largely unknown by local residents, and differs from the environment in which the tool was initially developed. Further work is needed to develop new tools or alter existing tools that can be adapted to diverse settings and cultures.

## Background

The early years of a child’s life are characterized by critical and rapid brain development and thus are the most effective time to help children reach their full potential [1]. Worldwide, children are unable to reach their full cognitive potential due to genetic, environmental, and psychological factors [2]. Global early child development (ECD) experts conservatively estimate that in developing countries, more than 200 million children under the age of five fail to reach their cognitive potential due to poverty, poor health, inadequate nutrition, and insufficient car e[2]. Early identification of and intervention with children who experience these factors are fundamental principles of child health [1]. Early cognitive and social-emotional development is predictive of school advancement in both developed and developing countries [2]. With increasing exposure to developmental risk factors, cognitive disparities increase and poor development becomes more established [2]. A study of South African children showed cognitive ability and achievement at the end of grade one predicted later school advancement [3].

Key developmental risk factors include inadequate cognitive stimulation, stunted growth, iodine deficiency, and iron-deficiency anemia [4]. Moreover, development is impeded in the presence of an array of risk factors including intrauterine growth restriction, malaria, lead exposure, enteric disease, maternal depression, exposure to violence, and HIV infection [4]. Sub-Saharan Africa has the highest prevalence of disadvantaged children under 5 years old (61%) in the worl d[2]. HIV infections in South Africa has also negatively impacted ECD for children of infected parents, increasing the risk of poor development; however home stimulation programs have been effective in improving cognitive and motor development in this population in South Afric a[5].

Developmental delays can be mitigated and reversed with focused early intervention s[2, 6, 7]. Interventions providing adequate cognitive stimulation and learning opportunities to young children resulted in better development of cognitive abilities and school readiness, regardless of risk condition, maternal resources, child gender, or countr y[1, 4, 8]. Studies from Bangladesh, China, India, and South Africa have shown that enhanced interactions between the mother and child through developmentally meaningful play improves cognitive development when delivered through home visits or counseling at health center s[4, 5, 9, 10]. Since development is malleable and manifests over time, ongoing monitoring is needed to identify children who seem on track at an early age, but may develop delays as they ag e[11].

There is a need to develop or adapt early child development tools for diverse populations; however, cross-cultural adaptations of these tools can be challenging [12]. Differences in language, culture, and resources between Western and non-Western contexts require culturally appropriate modifications in order to adapt pre-existing tools to new environments. To date, there is a knowledge gap related to specific modifications needed for non-Western populations to use Western screening tools, including those living in rural areas of sub-Saharan Africa. The purpose of this research study is to compare the adaptability and usability of two different tools used to detect developmental delay in children in rural South Africa. This research will contribute to the body of knowledge in ECD by disseminating the perceptions and expertise of local public health nurses about the usability of ECD tools in their community, and how these tools might be adapted to better fit their community’s needs. This research question developed out of a larger program and partnership with the Vhembe Health district in Limpopo, South Africa, with the overall goal of improving early child development in this region through implementing a early child development program. Two tools were chosen together by the authors and community partners: the Ages and States Questionnaire (a screening and monitoring tool) and the Cognitive Adaptive Test/Clinical Linguistic and Auditory Milestone Scale (an assessment tool).

The Ages and Stages Questionnaire (ASQ) is a screening tool used to identify children at risk for developmental delays, aged 2 months to 66 months [13]. The survey assesses five domains of development: gross motor, fine motor, communication, problem solving, and personal-social [13]. The survey requires a caregiver to report information about their child’s ability to complete developmental tasks, language acquisition, and social skills [13]. In previous ASQ validation studies, the ability to identify children with delay varied from 51%- 90%, depending on the age at the time of screening, with an overall sensitivity of 75% and specificity ranging from 81% to 92% [14].

The Cognitive Adaptive Test/Clinical Linguistic and Auditory Milestone Scale (CAT/CLAMS) was developed to identify global cognitive delay and language delay by evaluating language and problem-solving skills independently in children under 36 months old [15]. This assessment tool uses instruments (toys) to assess development while the evaluator directly observes the child’s performance [15]. The CAT/CLAMS test items are designed to naturally advance a child through the tasks until he or she is unable to complete a task [15]. This test has been shown to correlate well with other tools, and has high specificity of 95%-98% [14, 16, 17]. Researchers have measured a wide range of sensitivity, ranging from 30-45% in low birth weight infants in Taiwan to 85-96% for detecting cognitive delay in more general populations [14, 16, 18].

## Methods

This research study was reviewed and approved by the University of Virginia Institutional Review Board as well as by the Vhembe Health District of the Limpopo Health Department in South Africa. The purpose of this study was to compare the adaptability and usability of the ASQ or the CAT/CLAMS assessment tool to track ECD in the Vhembe Health District in Limpopo, South Africa. This district is primarily rural with small villages scattered throughout the countryside, and includes the small city of Thohoyandou. The Vhembe Health District consists of 112 clinics, 8 community health centers, and 6 public hospitals. Services at these centers are provided free or at low cost, however transportation in rural settings can be a barrier to care. Community health workers (locally referred to as “home-based carers”) are also used to augment health services by providing patient education and improving medication adherence in their communities. This research was conducted as part of a larger program, which aimed to improve the recognition and referral rates of developmental delays in young children in this region.

Although there are several different screening, monitoring, and assessment tools available to measure physical and cognitive development in young children, ASQ and CAT/CLAMS were selected based on the input and expertise of the local health contacts and the authors. The senior author of the study has experience using ASQ in the local community and CAT/CLAMS during pediatric exams. Local partners from South Africa visited the researchers in the United States prior to the research study and hypothesized that one of these tools may be a good fit in their home environment, and requested help comparing the two tools in Limpopo, South Africa, with the ultimate goal of adopting a tool.

Volunteer participants were recruited from nurses working in rural areas of the Vhembe Health District of Limpopo, South Africa. Nurses were chosen specifically as they are the primary community health providers in this region and are responsible for referring patients to physicians or hospitals as needed. They also lead the training for any lay community workers that serve their community and would be responsible for using the selected tool to identify at-risk children. Nurses were identified and recruited using flyers and a standard script following a referral from a local district nursing supervisor who was well connected within the health district. All participants were recruited through local contacts, and verbally consented to participate in English prior to the start of the focus group. In order to be eligible for the study, nurses had to be proficient in English writing and conversation, and work within the Vhembe Health District as a primary health clinic nurse or nursing student. All the nurses that volunteered met these criteria, and no participants were excluded or dropped out of the study prior to its completion. During the focus groups, refreshments were provided, however participants received no compensation for their participation. No non-participants other than the research team members were present during the focus groups.

Focus groups used open-ended, semi-structured questions, and were facilitated by a moderator, co-moderator and observer who were all female, and cultural outsiders. The focus groups discussed four pre-selected questions related to the usability and feasibility of each assessment tool, and follow-up questions were asked to further develop their thoughts and ideas. Two ninety-minute focus group sessions were conducted with local nurses using a semi-structured approach. Data were collected at one of the public primary health clinics within the community that the nurses practiced. Due to time constraints of participants, follow-up interviews or additional sessions were not able to be conducted with participants, and it is probable data saturation was not reached in this sample.

In total, eleven female nurses (eight professional nurses and three nursing students) were interviewed with nursing experience ranging from 0 – 28 years. Each focus group consisted of five to six nurses who were assigned into one of the two focus groups. Both focus groups were organized to include student nurses and professional nurses with a variety of experience. As the focus groups were the first meeting between the facilitators and participants, the focus group sessions began with group and facilitator introductions, followed by a twenty-minute educational session about the first of the two tools, and a twenty-five-minute discussion. This process was repeated, and each group learned and discussed the second tool and then compared the two tools. Using this procedure minimized any potential bias introduced as a result of the order in which the material was presented. The total time needed to learn and discuss each tool was 45 minutes, totaling ninety minutes for the session. Both educational sessions included a brief overview and demonstration of the assessment tool, a list of validated and prepared strengths and weaknesses, and time for questions and answers regarding the functionality of the tool.

Both focus groups were recorded and transcribed verbatim by members of the research team who were present during the focus group. Session notes, observations, and transcripts were reviewed and analyzed by the first author. Observations were used as supporting evidence when coding and categorizing data. The twenty-minute educational session was largely excluded from the analysis, unless there was discussion about the tool within the educational section. Each focus group was analyzed individually as well as combined with the concurrent session. Coupled data were coded using versus coding for further categorization and analysis. Versus coding was used to identify concepts and phenomena that were in direct conflict with each other and compare differences in responses between tools and across focus groups. The assessment tools were compared against each other, and codes were developed based on similarities and differences between the pairings. Codes were identified, defined, and categorized, with allowances for data to fall within multiple codes. Related codes were grouped into categories and clustered into themes based on shared ideas. The results from the analysis were shared, discussed, and agreed upon by the entire research team. Member checking was not performed.

## Results

After comparing comments from both groups in reference to either assessment tool, there were no relevant differences in opinion based on which tool was learned first or second. A total of 150 quotes relevant to the research question were identified from the transcripts of the two focus groups, and were coded into sixteen uniquely defined codes. These codes, their definitions, and an exemplar quote can be found in Table 1. These codes were grouped into six categories: current practice, usability, resource management, cultural adaptation, patient and parent factors, and new knowledge. A summary table of the strengths and weakness of each tool identified by the nurses in the focus group according to category is presented in Table 2. Two major themes, intrinsic cultural assumptions and inadequate knowledge of ECD within the community, emerged from the data analysis.

**Table 1:** Table of themes, categories, and codes identified by Group 1 and Group 2

Theme	Category	Code	Definition	Exemplar
Intrinsic Cultural Assumptions	Resource Management	Time Management	Describes how the tool will effect nurse’s time management in their daily practice	“We are always in a rush. It's not very practical for the clinic”
		Community Health Workers	Describes involvement or training of community health workers in assessment and implementation	“We're going to need more workers. We're going to need more people to be hired.”
		Financial Restrictions	Describes concerns related to costs of using the tool	“It will be a challenge buying.”
	Cultural Adaptations	Language Assumption	Describes language difference between English and their native languages	“If it's changed to say, Tsonga, then people around here might understand it.”
		Resource Assumption	Describes aspects of the tool that nurses cannot easily access	“We give them a stick and the children play with the mud and cup.”
	Patient and Parent Factors	Perceptions of Care	Nurse’s beliefs about the attitudes of parents when they visit the clinic	“Even the mothers, when they come to the clinic, they do not give themselves time for the clinic.”
		Parent-Child Perception	Nurse’s beliefs about how parents and children interact in the context of child development	“Children now a days, children are afraid of their parents.”
		Enhanced Patient Interaction	Describes improved patient care due to tool utilization	“I can not hurry to take the child to give medication, I can be able to first to see that this child is normal.”
Inadequate Knowledge of Child Development Within the Community	Current Practice	Current Practice	Describes current child health assessment and referral techniques	“And when we tried to play with her, she just stared. And when we called her, she don't respond. And then I refer her to my seniors. And then they referred her to the hospital … ”
		Health Knowledge Deficit	Describes known or perceived child health knowledge deficits of community members	“It is important for us to educated the home-based carer, to educate the people in the community to know the importance of child health in the community.”
	Usability	Setting	Describes or compares CAT/CLAMS or ASQ usability in different settings	“Once a person gets to understand perfectly it can be very practical, especially in [pediatric] wards. Here nurses, we are always looking at the time.”
		Scoring	Describes difficulty with scoring the tool	“I think that's the complicated part, the scoring.”
		Documentation	Describes difficulty with documenting child development assessment	“It is too much for the grannies, these old aged people will not be able to fill out the forms.”
		Usability	When nurses directly address the usability of the tool	“It's very easy and practical.”
	New Knowledge	New Knowledge	Describes skills or learning from training that can be used on patients	“Today I am learning so then after that, I can assess the sickness.”
		Secondary Outcomes	Describes benefits of assessment tool to areas other than child development	“I think it's going to help because they don't bring the child generally, they only bring the child when it is critically ill.”

**Table 2:** Strengths and Weaknesses of implementing the CAT/CLAMS and ASQ child development tool in Limpopo, South Africa^a^

	CAT/CLAMS		ASQ	
	Strengths	Weaknesses	Strengths	Weaknesses
**Usability**	**-** Calculates developmental score - Well suited for hospital or school environment - Feasible for use in clinic work flow	**-** Described as costly, time consuming, and difficult to calculate **-** Score calculation is moderately challenging - Better suited for low-volume days	**-** Described as easy, practical, uncomplicated, and simple - Uncomplicated administration - Visual graph to show where a child falls in relation to “cut off” scores - Identified as primary assessment tool - Feasible for use in clinic work flow	**-** Requires assessor to be literate - Score calculation is mildly challenging
**Resource Management**	None identified	**-** High cost of toys - Long administration time	- Low cost **-** Utilization of CHW - Minimal time commitment for nurses	**-** Cost of copying assessment surveys - Insufficient numbers of CHW to complete home assessments
**Cultural Adaptations**	None identified	- Moderate amount of translating needed - Expensive toys which are not all culturally appropriate	None identified	- Substantial amount of translating needed - Poor access to copiers and printers - Requires cultural modification of survey assessment questions
**Patient and Parent Factors**	- Direct observation of child’s abilities - Can use toys to teach mothers about meaningful play - Spend more time with patients - Nurses want to assess their own children using the tool	**-** Parents may not understand the importance of “play” during assessment and become impatient and leave - Child may be afraid of the nurse and underperform	- Assessment done in-home - Nurse can teach parents about results when they score the assessment tool	**-** Nurses distrust accuracy of information reported by parents

^a^The categories “current practice” and “new knowledge” are purposely omitted because there was no comparative data in either of these categories.

### Current Practice

According to the participating nurses, there is currently no system in place to routinely screen children for developmental delay in their community. Nurses stated that they use their own clinical judgment to identify children who may be delayed and refer them to a senior nurse for a second opinion, and eventually to the hospital for further testing. They also relied on family members, other clinicians in the community, or memories of their own children at specific ages.

The nurses in both groups also expressed frustration with the perceived indifference shown by the parents toward child health. Nurses explained that in their culture, parents will delay formalized care for their child after several weeks of illness, and ask neighbors and elders for advice or herbal remedies prior to seeking care at a clinic. According to the interviewed nurses, often by the time the child arrives in the clinic, he or she requires care above what is able to be provided at the clinic and must be transported to a hospital.

### Usability

When asked to compare the assessment tools, the nurses described the CAT/CLAMS tool as “costly,” “time consuming,” and “difficult to calculate” in comparison with the ASQ tool, which was described as “easy,” “practical,” “uncomplicated,” and “simple.” One of the nurses stated “I think ASQ is better than CAT/CLAMS because I can use it, it’s easier to use. The other one [CAT/CLAMS] is difficult to calculate but it is so nice to learn.” The nurses favored the straightforward, easy-to-read language of the ASQ tool, but noted that parents and grandparents may have difficulties completing the survey without the help of the trained community health worker due to language and literacy barriers. The nurses valued the knowledge resulting from the CAT/CLAMS tool, but reported it was difficult and impractical for nurses to use.

Calculating the results using either assessment tool was initially challenging for many nurses. Both groups identified scoring as the most difficult part of the tools, and required multiple explanations and demonstrations of how to properly score both assessments. The nurses generally felt more comfortable with the ASQ scoring by the end of the training session, but felt they could master the CAT/CLAMS scoring as well with more practice. Nurses liked reading the graphed scores for the ASQ and found them relatively easy to interpret after some practice. Although the CAT/CLAMS scoring was viewed as more difficult, the nurses valued knowing a calculated developmental age, and one nurse expressed wanting to use it on her own child: “I would really like a copy for my coming child so that I can be able to score him or her.”

When asked which tool they were more comfortable using, most nurses said they could use both tools, but thought that the CAT/CLAMS tool would be more appropriate for a different setting, where they would have more time to assess the child. Most nurses were reluctant to choose one tool over the other. Some suggested using CAT/CLAMS only on specific days in the clinic. “Once a person gets to understand perfectly [CAT/CLAMS] can be very practical, especially in [pediatric] wards. Here, nurses, we are always looking at the time.” The nurses brainstormed many ideas about the potential uses for the different tools in their community. In both groups, nurses thought CAT/CLAMS would be better suited for the hospital since they would have more time to administer it because the family would not expect to leave the hospital quickly. Some nurses suggested a program where ASQ would be assessed routinely in the community, and CAT/CLAMS on specific day or week in the clinic. “We will be using both, but this one [ASQ] we will be using mostly because CAT/CLAMS we will use it once … when we have that day for vaccines.” In the Vhembe Health District, there are periodic “vaccination days” where parents bring their child to be immunized, targeting the pediatric population. This would allow the nurses to screen many children all together on the same day, but does not allow for routine monitoring like ASQ.

### Resource Management

Limited time, finances, and resources were identified as barriers to implementing an ECD program using the CAT/CLAMS or ASQ tool. Both groups stated nurses would have to use their own money to print copies of the ASQ survey, or buy the toys for the CAT/CLAMS assessment. Some nurses viewed the CAT/CLAMS toys with interest; however, they perceived that only white children have the luxury of toys, because their parents have more resources and are able to afford expensive toys. “We are different because they [whites] get more money than us. They can buy the toys, many toys for their children. Young children. But with us, just give the baby stones.”

When implementing the ASQ screening tool, a major concern apart from printing was the increased staff needed to administer the surveys in the community. The nurses reported they would need to increase the number of community health workers (CHW) providing care in the community in order to serve the entire population. The nurses would also require additional time allocated for training and supervising the CHW. However, both groups of nurses were very supportive of the idea of using the CHW to share the workload of this program. The amount of time needed to administer the survey was a significant barrier to the use of CAT/CLAMS in the workplace. One group stressed time management while working in the clinic, however it was mentioned in both groups. The nurses reported their busy clinic environment prohibits fully assessing the child because they also need to tend to their other patients’ needs. Nurses also have competing priorities to consider including family planning, treatments, and many other tasks during their patient interactions.

We are the clinic. We are running the clinic. You do not get to play with the child and identify all of those [developmental milestones]. But at the hospital, they [hospital nurses] are the people who are used to be in the children’s ward. They can divide themselves more; you’re the one who will be doing the child. You do this. I will do this. But in the clinic facility, I do not think it will be possible.

### Cultural Adaptation

Considering both ASQ and CAT/CLAMS were developed for and initially tested within Western cultural contexts, it is not surprising that both tools would require significant language and cultural modifications to be applicable to those in Limpopo, South Africa. Both groups recommended translating the tools into the nurse’s or CHW’s native language. A nurse from group one requested, “Maybe you can change [ASQ] to Tsonga, because some of them, they can’t understand English but maybe if you transfer it to the other tongue it will be easier.”

Additional cultural modifications would be required for the language assessment and survey questions in both tools. For example, CAT/CLAMS refers to the child making “razzing” sounds, which the nurses did not understand for children starting to speak in that region. Children are also unfamiliar with many of the toys presented in CAT/CLAMS, which may impact the results of assessment. The nurses believed children would be distracted, attempting to understand the purpose of the toy rather than following the directions of the assessor. Nurses in both groups also expressed concerns accessing the toys needed for the assessment. For example, crayons are mentioned in both the ASQ and CAT/CLAMS assessment; however, children in this region do not scribble on paper or use crayons because they are cost prohibitive and not easily accessed. Instead, children play with sticks and draw in the mud. The ASQ also assesses fine motor development by asking the parents how well the child uses a spoon to eat. However, many people in rural Limpopo eat with their hands and do not use spoons.

### Patient and Parent Factors

The concept of parents playing with their children at an early age is unfamiliar to many of the families living in this region. Mothers often carry infants on their backs throughout the day and do not interact with the child in the same ways as Western mothers. The nurses speculated that parents may not understand why the assessment is being done in the clinic or in their home. Parents may not be able to answer many of the questions on the ASQ because they do not normally observe or interact with their children during play. When discussing CAT/CLAMS, nurses perceived that parents would not want to stay and wait for the assessment to be completed because many parents do not value the importance of play. The nurses were also skeptical that the information reported from parents would be accurate. In this instance, some participants favored CAT/CLAMS because they would be able to directly observe the child as he or she performs the requested tasks. However, many children fear women in nursing uniforms, which may make it harder for them to perform during the CAT/CLAMS assessment. For many children, the only time they see the nurse is when they are getting a vaccination, and their fear of pain may impede their ability to perform.

With both the ASQ and CAT/CLAMS tool, nurses believed they would be able to have a more meaningful interaction with parents and children in the clinic. With the ASQ tool, the nurses liked interpreting the survey and the ability to talk with the parent about the child’s developmental progress. One nurse mentioned that the CAT/CLAMS toys could be used to teach mothers about development during the assessment. Nurses could instruct parents to practice with their child to help the child develop in any areas that he or she may be delayed. CAT/CLAMS also requires spending more time with each patient, increasing the opportunity for parent education. In both groups, some nurses saw this as a positive attribute while others expressed many mothers may see the assessment as a waste of their time.

### New Knowledge

The nurses believed that the ECD assessment tools would improve the overall health of children in the community through improved surveillance. Nurses that were trained to use this tool would have new knowledge about ECD milestones, would obtain skills to assess development, and could identify child health problems earlier due to increased contact with patients through the developmental screenings. The nurses could teach the parents and community members the importance of play within the context of ECD. With the CAT/CLAMS tool, nurses learned about “toys” and games that can assess a specific developmental age and also be used as a teaching tool. With the ASQ tool, nurses could improve surveillance through more frequent contact and conversation with caretakers about a child’s well-being through the utilization of the CHW facilitating the ASQ assessment. One nurse stated, “It is important for us [nurses] to educate the home-based carer [CHW], to educate the people in the community, to know the importance of child health in the community.”

## Discussion

After conducting focus groups with two groups of nurses from this region, it was determined that an ECD monitoring program would be well received in this community. Based on the results of this study, the ASQ was identified as the most appropriate for this setting because it is more time efficient, and is less expensive than the CAT/CLAMS tool. However, nurses were reluctant to choose one tool over the other. This reluctance to choose could be culturally grounded or due to their inability to adequately test each tool in a natural clinical setting.

The nurse participants acknowledged the importance of ECD, but had limited experience or knowledge with formal developmental assessment or monitoring programs. They saw a monitoring program as a way to improve child health education within the community, to assess their own children, and increase child health surveillance in the community with the utilization of community health workers in the home. In a similar study that assessed the usability of the social-emotional section of the ASQ questionnaire in Malaysia, nurses found it easy to implement an effective measure for screening young children for social-emotional problems, and training improved nurses’ knowledge and attitude toward ECD [19].

One of the weaknesses of the ASQ tool was a distrust of the accuracy of the parental reports. However, a recent study has shown parent-completed screening questionnaires can be as accurate as those performed by a health provider. Despite variations in socioeconomic status, geographic location, or parental well-being, parents are able to give accurate information about their child’s development [14]. However, this requires further exploration as it is unknown if these findings would be be applicable to Limpopo due to vast differences in language and cultural practices.

Implementing the ASQ would also require significant training and diligence from parents and CHW. Although the CHW are seen as respected and knowledgeable members of their communities, they have no formal training in ECD, and would need instruction to assist the parents with the ASQ survey. Parents would also need to spend significant amounts of time observing and playing with their child, as well as completing and returning the survey several times throughout the child’s development. This may not be feasible without first educating the parents about ECD, which would require significant time and resources from the rural clinics.

Parental illiteracy was also a concern among the nurses; however, previous studies have addressed this barrier through oral administration of the tool and with the use of CHW assisting with in-home survey administration [14]. Home-based early intervention programs have been successful in other low-resource areas. In a study of high-risk infants in India, in-home early intervention programs were more likely to reach high-risk infants compared with those administered in a health center [6]. A major strength of the ASQ tool identified by the nurses was the parental involvement required in assessing the child. Collaborating with parents helps them understand how assessment and play could improve their child’s development. This concept is also supported in recent literature. When parents are provided with opportunities to observe, record, and learn about their child, they can better understand meaningful accomplishments and appreciate their child’s efforts, successes, and achievements over time [20]. However, it is unknown if these findings are applicable to Limpopo due to cultural differences in parent-child interactions.

In a survey of American primary care practitioners, the majority (82%) stated that time constraints were the largest barrier to administering screening tests [14]. The participating nurses also identified time as a major concern, especially with respect to the CAT/CLAMS tool. The other major concern was the cost of the program. In the United States, parent-report measures ($11-$17USD) were more cost effective than screening tests administered by health professionals ($22-$82USD) [14]. However, these estimates may not be transferrable to the population in Limpopo due to considerable differences in health care delivery. The nurses also mentioned that children might be afraid to perform during the CAT/CLAMS assessment, which is supported in the literature [14].

Program adherence may also be a barrier to implementation, however it was not mentioned by the participants. Parents would need to fill out several surveys throughout their child’s first three years, and attrition may be a limitation in identifying developmental trends in individual children. Although nurses did state the parents might not want to wait for the CAT/CLAMS assessment to be completed in the clinic, the nurses did not speculate as to how parents may receive the ASQ assessments in the home. In India, adherence to an early child intervention was only 59.2% for three or more sessions when children were brought to a clinic for assessment [14]. Although the program assessment would be in-home, parents would need to bring their child and the survey to the clinic for interpretation. Barriers such as limited transportation to the clinic, unavailable childcare, and inability to get time off of work may affect parental adherence to a child monitoring program in this region.

The small sample of nurses and unique population limits the generalizability of the results of this study. It is also unlikely that data saturation was reached given the short amount of time the nurses were able to learn and use each tool, the small size of each group, and the single time-point for the interviews. However, the data found in this study is validated by other similar studies of diverse populations that were discussed earlier in this section. The focus groups were also conducted by “cultural outsiders” and in English, which was not the native language of the participants and could lead to misunderstandings. To address this limitation, researchers took measures to clarify and restate questions and responses to ensure adequate understanding.

There are also some limitations associated with the choice and comparability of the tools. The ASQ is designed as a screening or monitoring tool used by caregivers and shared with health care providers. The CAT/CLAMS is an assessment tool used by health care providers in a practice setting. The preference for the ASQ may reflect a preference for a screening or monitoring tool over an assessment tool such as CAT/CLAMS, and not a preference for the tool itself.

There are also challenges with using a screening or monitoring tool for monitoring in contexts with limited expertise or resources. Screening measures are often not comprehensive and may not be uniformly sensitive to change over time or to the impact of developmental interventions. These screening items were selected to identify children that are under performing, and the screening tool may not accurately reflect change over time or positive progress for children with above average development [21].

There are opportunities for future research in Limpopo around ECD. The usability and generalizability of the ASQ tool needs to be further assessed in this population. More nurses would need to be trained in ECD screening and to use the ASQ tool. The ASQ tool would also need to be culturally adapted and validated for this population. Additionally, CAT/CLAMS instruments could be adapted as well for this community and tested in hospitals. A longitudinal study using a small sample of children in the region would show if the ASQ tool is useful for identifying and tracking developmentally delayed children.

## Conclusion

The ASQ is a more feasible option for ECD assessment than the CAT/CLAMS tool; however, both tools would require significant training, resources, and commitment from the community in order to be properly implemented. One major advantage of the ASQ is that community health workers can administer it in the home. This leads to increased community investment and knowledge dissemination about the importance of ECD beyond the primary health nurses. This also increases contact between new mothers and a health advisor, which could potentially improve disease surveillance and prevention in this population. The ASQ involves parental participation in assessing and improving their child’s development, and teaches parents different developmental milestones their child has or will achieve. Parents would have an active role in assessing their children and can learn about ECD through the assessment process. It also requires less time in the clinic with the nurse, decreasing the burden on the nurses and sharing the program responsibilities with the community health workers.

This research can help inform others of the challenges and considerations related to adapting a Western tool to a non-Western context. Although not comprehensive, our findings are supported by other findings in the literature, and are informative for others doing early child development work in this population. Once culturally modified, these tools can help identify developmentally delayed children in regions where the services are not commonly available. This study is the first to investigate ECD tools in rural Limpopo, and supports the conclusion that ECD monitoring is feasible and beneficial in rural Limpopo, South Africa. This research may help to improve child health in this region through developmental tracking and parental education. These changes could result in long-term improvements in child health.

## Supplementary information

**Additional file 1.** Focus Group Interview Guide

## Data Availability

The interview transcripts generated and/or analyzed during the current study are not publicly available due protecting privacy, but are available from the corresponding author on reasonable request.

## Abbreviations

ASQ: Ages and States Questionnaire
CAT/CLAMS: Cognitive Adaptive Test/Clinical Linguistic and Auditory Milestone Scale
CHW: Community Health Workers
ECD: Early Child Development
HIV: Human Immodeficiency Virus
USD: United States Dollars
