# Supplementary material for: Comparing Two Early Child Development Assessment Tools in Rural Limpopo, South Africa
Source: BMC Pediatr. 2020 May 7;20:197. doi: 10.1186/s12887-020-02101-0 (PMC7204218; doi:10.1186/s12887-020-02101-0)
Supplement: Supplementary file 1 — Additional file 1. Focus Group Interview Guide [file 12887_2020_2101_MOESM1_ESM.docx]

Semi-Structured Interview Guide

1. What did you like about the [Ages & Stages or CAT/CLAMS] tool?
2. What was difficult or challenging about the [Ages & Stages or CAT/CLAMS] tool?
3. How confident do you feel using [Ages & Stages or CAT/CLAMS] on a child in your clinic?
4. How do you see [Ages & Stages or CAT/CLAMS] fitting into your work with young children in the clinics?
5. Which assessment tool, CAT/CLAMS or Ages and Stages seemed like it would best fit into your practice? Why?
6. Is there anything else you would like to say about either assessment tool?
